# Cerebrotendinous xanthomatosis tremor successfully controlled post-ventral intermediate nucleus-deep brain stimulation: a case report

**DOI:** 10.3389/fneur.2023.1243379

**Published:** 2023-08-30

**Authors:** Alyson M. Rich, Ema V. Karakoleva, James McInerney, Elana Farace, Sol De Jesus

**Affiliations:** ^1^Department of Neurology, Penn State College of Medicine, Hershey, PA, United States; ^2^Department of Neurosurgery, Penn State Health-Hershey Medical Center, Hershey, PA, United States

**Keywords:** cerebrotendinous xanthomatosis (CTX), deep brain stimulation (DBS), tremor, hyperkinetic movement disorder, lipid storage disease

## Abstract

Cerebrotendinous xanthomatosis (CTX) is a rare autosomal recessive disorder caused by a deficiency of the sterol 27-hydroxylase enzyme. This deficiency results in excess production and accumulation of cholestanol, which can lead to many clinical findings within the first three decades of life, including progressive neurological dysfunction. This is a treatable condition with improvements in neurological and non-neurological symptoms upon the early initiation of replacement therapy. This case report details a 42 years-old left-handed male in whom deep brain stimulation (DBS) intervention was pursued due to a limiting tremor related to delayed diagnosis and treatment of CTX at 22 years old. The application of DBS in treating tremors in a CTX patient has not previously been reported. For our patient, application of DBS led to meaningful and longstanding tremor control benefits that have required minimal changes to stimulation parameters post-DBS. These improvements to tremor were achieved without negative impact to his other CTX related comorbidities.

## 1. Introduction

Cerebrotendinous xanthomatosis (CTX) is a rare autosomal recessive disorder caused by a deficiency of the sterol 27-hydroxylase enzyme (1). This deficiency results in excess production and accumulation of cholestanol in multiple tissues, including the eyes, tendons, and brain (1). The excess accumulation can lead to early-onset cataracts, tendon xanthomas, and progressive neurological dysfunction. These clinical signs typically begin manifesting in early infancy and are more notably present within the first three decades of life (2, 3). Treatment of CTX with replacement chenodeoxycholic acid (CDCA) can prevent development of irreversible neurological and non-neurological symptoms and/or stabilize symptoms if initiated early (4). However, disease progression has been observed in patients initiated on CDCA replacement beyond the age of 24, making early diagnosis and replacement therapy critical (5–7). Neurologic symptoms that can develop—including epilepsy, spasticity, and a variety of hypokinetic and hyperkinetic movement disorders thought to be a late disease manifestation—are treated symptomatically (3). Deep brain stimulation (DBS) is an established therapy option for patients with various movement disorders and has been applied to less common tremor disorders (8–12). However, the tolerability and response of DBS in treating tremor in a patient with underlying CTX has not previously been reported. This case report details a single case of CTX-tremor that was successfully treated with unilateral thalamic ventral intermediate nucleus (VIM) DBS.

## 2. Case description

The patient is a 42 years-old left-handed male with multiple CTX-related sequalae including developmental delay, cataracts, epilepsy, diabetes, chronic pancreatitis, anxiety, depression, parkinsonism, and postural/action tremor. CDCA replacement therapy, taken 250 mg orally three times a day, was delayed due to his CTX remaining undiagnosed until he was 22 years-old. His diagnosis and CTX management occurred at an outside institution based on elevated cholestanol levels, brain imaging findings as detailed below, and subsequent genetic testing that were not available to us upon his transfer of care at our institution. Onset of movement symptoms predominantly impacting his left hemibody have been present at least from the mid-2000s. He reported dramatic worsening of tremor in 2016 in the setting of long-standing use of aripiprazole since 2010 for mood stabilization without any recent adjustments. There was a transient initial improvement to tremor and parkinsonism with levodopa (carbidopa-levodopa IR 25 mg–100 mg 1 tablet with carbidopa 25 mg taken orally four times a day, ondansetron 8 mg taken orally 30 min prior to carbidopa-levodopa) and amantadine (100 mg 1 tablet three times a day); however, he subsequently developed gastrointestinal side effects with attempts at further titration. Trials with primidone and topiramate were unsuccessful and also led to intolerable side effects, and bradycardia limited the use of beta blockers.

**Figure 1:**
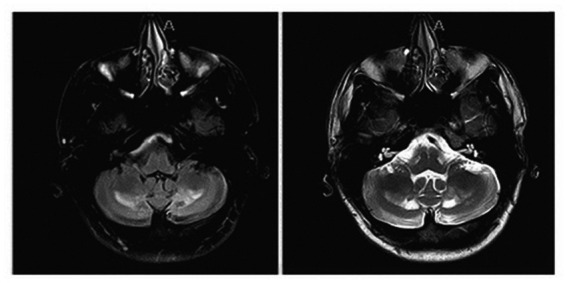
Sequela of known cerebrotendinous xanthomatosis on brain MRI: non-contrast MRI showing a nonspecific finding of diffuse increase in signal intensity of the white matter in the bilateral dentate on FLAIR weighted imaging (left image) and T2 (right image) sequences.

On evaluation, his unified Parkinson’s disease rating scale (UPDRS) part 3 OFF score was 37/108 and ON score was 31/108. Additionally, the tremor rating scale (Fahn–Tolosa–Marin) pre-DBS total score was 32/144 (moderate functional disability). On examination he exhibited left predominant rest tremor, rigidity, bradykinesia, gait changes without postural instability with additional findings of a primarily left upper extremity action/postural tremor. His main limitations subjectively reported were due to action/postural tremor impacting his dominant hand. Formal neuropsychological testing revealed baseline deficits in verbal memory (immediate recall 1st percentile) with delayed recall and recognition in the average range. Executive function was significantly impaired (Trails A 1st percentile, Trails B discontinued) with categorical fluency in the borderline range (5th percentile). Baseline physical therapy (PT) evaluations revealed a BERG balance scale 51/56 (range 0–56 with scores above 41 indicating independence with ambulation without assistance), timed-up-and-go 9.3 s (older adults who take longer than 14 s have an increased risk of fall), 5 times sit to stand 12.10 s (±15 s = risk of fall). A non-contrast MRI of the patient’s brain at the age of 21, around the time of worsening of symptoms, demonstrated nonspecific findings of a diffuse increase in signal intensity of the white matter in the bilateral dentate on T2 and FLAIR weighted imaging persistent in his planning MRI obtained in 2018 (Figure 1).

The patient was counseled regarding the lack of evidence regarding the role of DBS to treat tremor in individuals with CTX. Given the extensive impact on his quality of life due to his left-sided tremor affecting his dominant side, the patient and his care team elected to proceed with surgery.

He underwent a unilateral right-sided Medtronic DBS lead placement to the VIM of the thalamus, followed by a right anterior chest wall placement of an Activa primary cell implantable pulse generator (IPG) 1 week later. He reported a “honeymoon” (lesion) effect of 75%–80% prior to turning on stimulation at initial programming session. Threshold evaluation was conducted at 90 pulse width (PW) and 130 Hz frequency, with noted improved tremor at lower voltages and more ventral contacts. Final initial settings 2-C+ 2.0v 90PW 130 Hz.

## 3. Results—follow up and outcomes

### 3.1. Initial and short-term effects

At the initial programming visit prior to turning on stimulation his tone was mildly increased bilaterally at baseline and there remained a subjective and objective reduction in tremor from suspected lesion effect (tremor remaining in left hemibody rest tremor 1/4, postural/action tremor 1/4, leg tremor 1/4, all other measures 0). Although patient had discontinued amantadine and carbidopa-levodopa on his own for concerns of GI side effects prior to initial programming session, he remained on propranolol 10 mg 1 tablet TID for concomitant diagnosis of portal hypertension. By 1 month visit post initial programming signaling short-term effects, he reported symptom improvement of his LUE tremor of 90% (tremor remaining in left hemibody rest postural/action tremor 1/4, all other measures 0) and reported no changes in mood, cognition, speaking, or swallowing. His 3 months post-operative neuropsychological performance was stable compared to his baseline performance. However, he did require seeing PT after the first month for concerns of imbalance with noted minimal changes to BERG balance scale 48/56, timed-up-and-go 11.5 s, 5 times sit to stand 12.28 s. He was optimized after the initial programming session and remained with settings unchanged over the first 6 months period. Post-DBS tremor rating scale improved to a total score of 15 with previous score of 32 and marked improvement (50%–100%) on subjective assessment by the patient compared with his last visit.

### 3.2. Complication

Unfortunately, the patient developed complications of right cranial and infra-auricular wound breakdown and presented to the OR for a wound revision 7.5 months after his DBS lead insertion surgery. He experienced another wound breakdown with serosanguineous drainage over the IPG incision site at the right anterior chest wall and underwent a second wound revision surgery 11 months after the initial IPG placement. Ultimately these wound revisions failed, and the patient underwent removal of the unilateral right DBS lead of the IPG 1 year after their original insertion. Following a 2 weeks course of postoperative intravenous ciprofloxacin 250 mg twice a day, he recovered well.

The patient’s left-sided tremor re-emerged approximately two and a half weeks after DBS removal and he continued to experience issues, mainly with feeding himself and stuttering speech. According to the patient’s mother, who is his primary caregiver, his tremors remained mildly improved from prior to DBS. These symptoms were significantly distressing to the patient, and he elected to proceed with DBS re-implantation.

### 3.3. DBS re-implantation

Three months after wound recovery, he underwent a fiducial screw implantation, a re-implant of his right unilateral DBS targeting the VIM, and an Activa SC IPG to the right anterior chest wall. His stimulation (2-C+ 2.0v 90PW 130 Hz) was initiated 1 day post-procedure, unchanged from his initial programming session. Three months post-implantation, the patient reported significant improvement of his tremor and being able to eat and write using his left hand, as well as improvement of his speech, memory, and balance with some unsteadiness while going down hills. Post-DBS tremor rating scale total score was not available.

### 3.4. Long-term effects

The patient has continued follow-ups with his interdisciplinary team, including his primary care provider, neurology, endocrinology, psychiatry, and ophthalmology. Two years after re-implantation of the right VIM lead, tremor symptom control remains very much improved (tremor remaining in left hemibody rest postural/action tremor 1/4, all other measures 0). He remains on propranolol 10 mg TID for his portal hypertension. While adjustments were made to the active electrode, overall settings have remained relatively stable (1-C+ 2.0v 90PW 135 Hz). He completed PT in April 2022, at which time he had a BERG balance scale 52–53/56, timed-up-and-go 9.70 s, 5 times sit to stand 14.67 s, which are all comparable to pre-DBS assessments except for increase in sit to stand time. Although he notes intermittent stuttering speech and gait changes, his most current neurology assessments as of October 2022 include a Fahn–Tolosa–Marin tremor rating of 6/80 on medication. His parkinsonian symptoms of bradykinesia and rigidity have remained largely unchanged in the setting of VIM stimulation. Due to worsening depression disorder and anger issues, patient’s mood medications were adjusted, and he has remained well controlled. Overall, the patient’s tremors remained stable with very good functional ability including eating, drinking, cutting food, using buttons and zippers. For his stutter, he completed speech therapy with good improvement of his speech, but he has recently had worsening of his stutter and is considering returning to speech therapy.

## 4. Discussion

**Figure 2:**
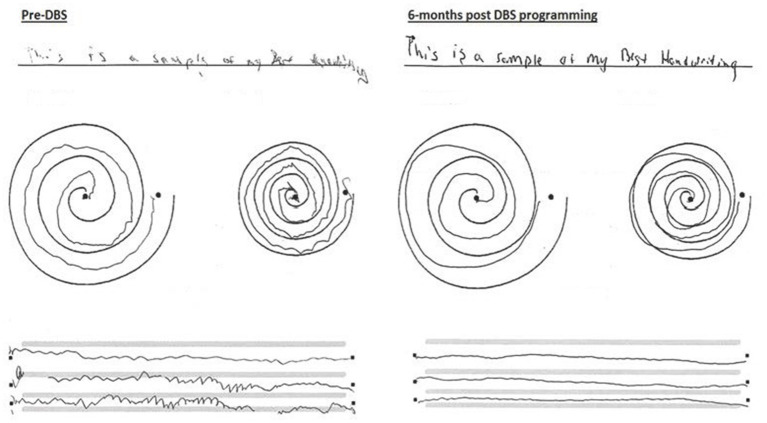
Handwriting and drawings of dominant left upper extremity: motor examination pre-DBS placement and 6 months post-DBS VIM placement.

Although the predictive value of the levodopa challenge score on parkinsonian symptoms control with DBS implantation is well documented (13) it was unclear whether the patient would benefit from the procedure given his mixed rest and action-postural tremor alongside a poor levodopa challenge score. Focusing on the limiting tremor symptoms impacting his dominant hand, meaningful improvements were achieved. Handwriting was found to improve in approximately 70% of patients following DBS implantation targeting the VIM (14). This patient’s handwriting was found to improve significantly during DBS programming, exhibiting improved legibility and reduced tremor post stimulation (Figure 2). As medication regimen remained unchanged pre- and post-DBS programming, the observed effects on symptom control were attributed to VIM-targeted stimulation and less to pharmacologic control, although synergistic effect with ongoing beta blocker cannot be entirely ruled out.

Our patient had MRI changes involving the cerebellar dentate nucleus that may explain in part the movement disorder manifestations and in particular his mixed tremor symptoms. Although the precise mechanisms by which VIM-DBS influences tremor remain unknown, advances in technology and focus on neuronal synchrony as well as diffusion tractography have further emphasized the role of the cerebellothalamocortical pathway in tremor contribution (15–17). The association between cortical-subcortical pathways impacted by VIM-DBS stimulation in relation to CTX pathophysiology remains to be elucidated.

Possible side effects from DBS targeting the VIM include dysarthria, impaired gait and balance, and cognition difficulties, with more pronounced effects observed following bilateral lead placement (18). Although the patient did report stuttering and gait disturbances after DBS implantation, therapy significantly helped improve his speech and gait. Objectively, gait measures did exhibit some fluctuations but remained overall stable. Due to the patient’s history of behavioral and cognitive issues prior to DBS therapy, there was hesitation on the part of the providers to proceed regarding tolerability of the DBS procedural process as well as stimulation. However, neuropsychological assessment offered insight into his baseline neurocognitive performance and helped provider confidence regarding his healthy social support system and his understanding of the risks and benefits of the DBS. Importantly, he did not exhibit a decline or worsening from a neuropsychological standpoint post-DBS. Continued monitoring by a multidisciplinary team has ensured patient safety.

While he did experience infectious complications requiring wound care, this is a risk in any surgery, including DBS implantation (19–21) and he opted for re-implantation given the benefits achieved from his initial procedure. While CTX is a rare and treatable disorder that should not be missed given promising improvements and stability of symptoms with early intervention, this report highlights the application of DBS in a case where a delay in treatment of CTX led to limiting tremors that were safely and effectively managed with DBS.

## 5. Conclusion

This gentleman is the first case known to the authors demonstrating that DBS can be successfully used to treat tremors secondary to CTX. While in general, CTX patients stabilize in their course once on replacement therapy with CDCA, factors such as age of initiation, duration, and adherence to medication regimen can affect treatment efficacy and risks of developing complications (5). It is therefore imperative that a multidisciplinary team of neurologists, ophthalmologists, and metabolic specialists are available to monitor the comprehensive health of these patients, especially in cases of delayed onset of replacement therapy such as in this case.

## Data availability statement

The original contributions presented in the study are included in the article/supplementary material, further inquiries can be directed to the corresponding author.

## Ethics statement

Ethical approval was not required for the study involving humans in accordance with the local legislation and institutional requirements. Written informed consent to participate in this study was not required from the participants or the participants’ legal guardians/next of kin in accordance with the national legislation and the institutional requirements. Written informed consent was obtained from the individual(s) for the publication of any potentially identifiable images or data included in this article. Written informed consent was obtained from the participant/patient(s) for the publication of this case report.

## Author contributions

AR and SDJ contributed to the conception of the case report. EK organized patient data. EK and AR wrote the first draft of the manuscript. SDJ edited and wrote sections of the manuscript. JM and EF were directly involved with patient care and edited sections of the manuscript. All authors contributed to manuscript revision, read, and approved the submitted version.

## Conflict of interest

SDJ has received support unrelated to this research for her role as an Educational Consultant for Medtronic Inc.

The remaining authors declare that the research was conducted in the absence of any commercial or financial relationships that could be construed as a potential conflict of interest.

## Publisher’s note

All claims expressed in this article are solely those of the authors and do not necessarily represent those of their affiliated organizations, or those of the publisher, the editors and the reviewers. Any product that may be evaluated in this article, or claim that may be made by its manufacturer, is not guaranteed or endorsed by the publisher.
